# Effect of coffee solution on colour stability of various clear aligner attachment composites, does surface sealing make a difference?: An in vitro study

**DOI:** 10.1186/s12903-026-09018-7

**Published:** 2026-06-29

**Authors:** Sadaf Azadi Çalışkan, Ismail Ata Orgun

**Affiliations:** Department of Orthodontics, Faculty of Dentistry, Cyprus Health and Social Science University, Mersin, Turkey

**Keywords:** Attachment, Colour stability, Clear aligner, Resin composite

## Abstract

**Background:**

In this exploratory study is the first to test the efficacy of a surface sealant in preventing discolouration of orthodontic attachments. It aimed to evaluate the colour stability of four different resin composites used in orthodontic attachments after immersion in a coffee solution and to investigate the effect of surface sealant application on their colour stability.

**Methods:**

A total of 96 specimens were prepared using four resin composites: Aligner Connect, Estelite Sigma Quick, Universal Flo and Transbond XT (*n* = 24 per group). Each resin composite group was divided into two subgroups (*n* = 12); one received a surface sealant (Permaseal, Ultradent), and the other served as a control. All specimens were immersed in a coffee solution and colour measurements were recorded at three simulated time intervals: T1 (1 month), T2 (3 months), and T3 (6 months). Colour differences (∆E₀₀) were calculated using the CIEDE2000 formula. Statistical analysis was performed using repeated measures 2-way ANOVA followed by Tukey’s post-hoc test (α = 0.05).

**Results:**

All resin composites showed a statistically significant increase in discolouration over time (*p <* 0.05). The Transbond XT group demonstrated the highest ∆E₀₀ (13.57 ± 0.77) values on Day 6, indicating the greatest level of discolouration. All resin composites showed ∆E₀₀ values exceeding the clinically acceptable threshold of 1.8.

**Conclusion:**

Exposure to coffee solution caused clinically unacceptable discolouration in all tested attachment resin composites. The type of resin composites played a critical role in determining the degree of discolouration in orthodontic attachment fabrication. Application of the surface sealant (Permaseal) did not provide a clinical advantage in improving resistance to discolouration form the coffee solution.

## Background

Over the past two decades, advancements in orthodontic materials and digital techniques have led to the development of clear aligners as a widely adopted alternative to conventional fixed appliances [1, 2]. They offer greater patient comfort, improved oral hygiene, and enhanced aesthetics, owing to their removability during eating and cleaning, which support periodontal health [3, 4].

To achieve optimal tooth movement with clear aligners, auxiliary components such as elastics, power ridges and attachments to refine force application [5–7]. Attachments enhance biomechanical control and aligner retention by modifying force vectors. Their position and shape are digitally planned, and a transparent guide tray is fabricated to transfer attachments intraorally. After prefilling the tray reservoirs with resin composite, the tray is seated, and the material is light cured through the tray [8]. Both paste-like and flowable resin composites are used; flowable types offer easier handling, whereas paste-like types provide better mechanical strength [8–10]. However, beyond mechanics, colour stability is also critical, as these composites must resist discolouration throughout the treatment [11]. Resin composites are prone to staining due to intrinsic and extrinsic factors such as temperature change, water sorption and pH fluctuations [12–14]. Given that aligner therapy can last up to two years, maintaining colour stability is clinically important.

Unlike restorative composites, orthodontic attachments are not polished after curing [15, 16]. When cured against a polyester (Mylar) surface, composites form a resin-rich outer layer that absorbs water and becomes more susceptible to staining [17]. Consequently, attachments fabricated this way may be inherently more prone to discolouration. Since many patients choose aligners for aesthetic reasons, visible staining of attachments can reduce patient satisfaction [18].

In restorative dentistry, surface sealants have been developed to increase the resistance of composite restorations to discolouration and to enhance their surface properties. These lightly filled or unfilled low-viscosity sealants infiltrate micro-porosities by forming an ultra thin protective layer. As a result, water absorption decreases, leading to enhanced colour stability and improved sealing thereby reducing microleakage [19, 20]. While several studies have reported favorable structural and esthetic outcomes following the application of surface sealants on resin composites [21, 22], others have questioned their clinical significance [23, 24].

Despite increasing interest in the aesthetic performance of materials used for orthodontic attachments, current evidence on their colour stability remains limited. In particular, the potential effect of surface sealant application on the discolouration of orthodontic attachments -structures that are clinically prone to staining due to their unpolished, resin-rich surfaces- has not yet been investigated. This represents a clinically significant gap, especially in light of the growing demand for aesthetic orthodontic treatment. Therefore, this study aimed to evaluate the colour stability of different resin composites used in the fabrication of orthodontic attachments, including both orthodontic and restorative materials, and to determine whether the application of a surface sealant enhances their resistance to discolouration.

This preliminary study aimed to evaluate the colour stability of resin composites used for different clinical purposes (2 orthodontic resin composite, 2 restorative resin composite) in the fabrication of orthodontic attachments and to determine whether the application of a surface sealant enhances their resistance to discolouration.

The null hypotheses tested were as follows:

1. Surface sealant application would not significantly improve the colour stability of any resin composite.
2. There would be no significant difference in colour stability between orthodontic and restorative resin composites.

## Methods

A priori power analysis was performed using G*Power 3.1.9.6 (Düsseldorf, Germany) for a repeated-measures ANOVA (within–between interaction) [25]. Assuming a medium effect (f = 0.25) [26], α = 0.05, power = 0.95, and a non-sphericity correction (ε = 0.75), the required total sample size was 96 specimens (8 groups of 12 specimens). The interaction between composite type and surface treatment was used as the primary effect for power estimation, as it represented the main research hypothesis.

### Experimental groups

For the experiment, 4 primary groups were established with different light-cured resin composites used for fabricating attachments in clear aligner therapy: one nano-hybrid paste-like restorative composite (Estelite Sigma Quick, Tokuyama, Tokyo, Japan), one high-filler flowable restorative composite (Universal Flo, GC, Tokyo, Japan), one orthodontic adhesive (Transbond XT, 3 M Espe, Monrovia, USA) and one injectable aligner composite (Aligner Connect, GC, Tokyo, Japan), which is specifically developed for attachment fabrication (Table 1).

**Table 1:** Compositions of the dental resin composite materials used in this study

Commercial Name	**Colour**	**Composite Type and Classification**	**Filler System**	**Manufacturer**
GC Aligner Connect	Standard	Injectable Orthodontic Composite	Octahydro-4,7-methano-1H-(indenediyl) bis (methylene) bismethacrylate, 1,3,5-Triazine-2,4,6- triamine, polymer with formaldehyde, 2,2'- ethylenedioxydiethyl dimethacrylate, 2-(2H- benzotriazol-2-yl)-p-cresol, UDMA	GC Corporation, Tokyo, Japan
G-aenial Universal Flo	A2	Flowable Restorative Composite	BIS-EMA, UDMA, TEGDMA, bis-MEPP, silicon dioxide, strontium glass (10–200 nm)	GC Corporation, Tokyo, Japan
Estelite Sigma Quick	A2	Paste-like Restorative Composite	BIS-GMA, TEGDMA, spherical submicron filler mean particle size: 0.2 particle size range: 0.1–0.3	Tokuyama, Tokyo, Japan
Transbond XT Light-cure Adhesive	Standard	Orthodontic Adhesive	Silane treated quartz, Bis-GMA, Bisphenol A Dimethacrylate, Silane treated silica, diphenyliodonium hexafluorophosphate, Triphenylantimony	3 M Unitek, Monrovia, USA
Permaseal	-	Restorative Surface Sealant	Bis-GMA 60%, TEGDMA 40%, 1-dimethylaminoethyl metacrylate < 3%	Ultradent Products, South Jordan, USA

*Bis-GMA* Bisphenol A-glycidyl methacrylate, *TEGDMA* Triethylene glycol dimethacrylate, *UDMA* Urethane dimethacrylate, *Bis-EMA* Bisphenol A- ethoxylated dimethacrylate, *PEGDMA* Polyethylene glycol dimethacrylate

Each primary group was subsequently subdivided into two sub-groups: one in which a surface sealant (Permaseal, Ultradent, South Jordan, USA) was applied and one in which no sealant was applied. In the sealant-applied subgroups, specimens were prepared and then the surface sealant agent was applied. In the no-sealant subgroups, which served as the self-control groups, no additional procedures were performed (Table 2).

**Table 2:** Group assignment of the study (*n* = 12)

Composite type	**Product**	**Surface**	**Group name**
Injectable aligner composite	Aligner Connect	No-seal	AL-no seal
		Seal	AL-seal
Flowable restorative composite	Universal Flo	No-seal	FL-no seal
		Seal	FL-seal
Paste-like restorative composite	Estelite Sigma Quick	No-seal	SG-no seal
		Seal	SG-seal
Orthodontic adhesive	Transbond XT	No-seal	XT- no seal
		Seal	XT-seal

*AL* Aligner Connect, *FL* Universal Flo, *SG* Estelite Sigma Quick, *XT* Transbond XT

### Specimen preparation

For attachments, disk-shaped specimens, each with a diameter of 7 mm and a thickness of 1 mm were fabricated. For eight groups, total twenty-four B1 shade zirconia (KATANA™ Zirconia UTML, Kuraray Noritake, Japan) blocks (36 mm wide, 4 mm thick, 9 mm deep) were fabricated to provide a standardized background, to prevent reflections from the underlying surface during colour measurements and to simulate the clinical substrate to which the attachment will be attached [27].

Randomly selected four attachment specimens were bonded onto each block with an adhesive system (Clearfil SE Bond, Kuraray, Osaka, Japan), ensuring equal spacing between them. Prior to bonding, the zirconia surfaces were air-abraded with 50 μm alumina (0.23 MPa pressure, 5 mm distance, 90° angle) using an intraoral sandblaster (Microetcher IIA, Danville Materials, S. Ramon, CA, USA) [28, 29]. Following sandblasting, the surfaces were cleaned with 37% phosphoric acid for 20 s, thoroughly rinsed with water, and air-dried. Subsequently, the primer of the adhesive system was actively applied to the zirconia surface and left undisturbed for 20 s, then gently air-dried. Thereafter, the bonding agent was applied, air-thinned to obtain a uniform film, and light-cured for 10 s in accordance with the manufacturer’s instructions.

**Fig. 1:**
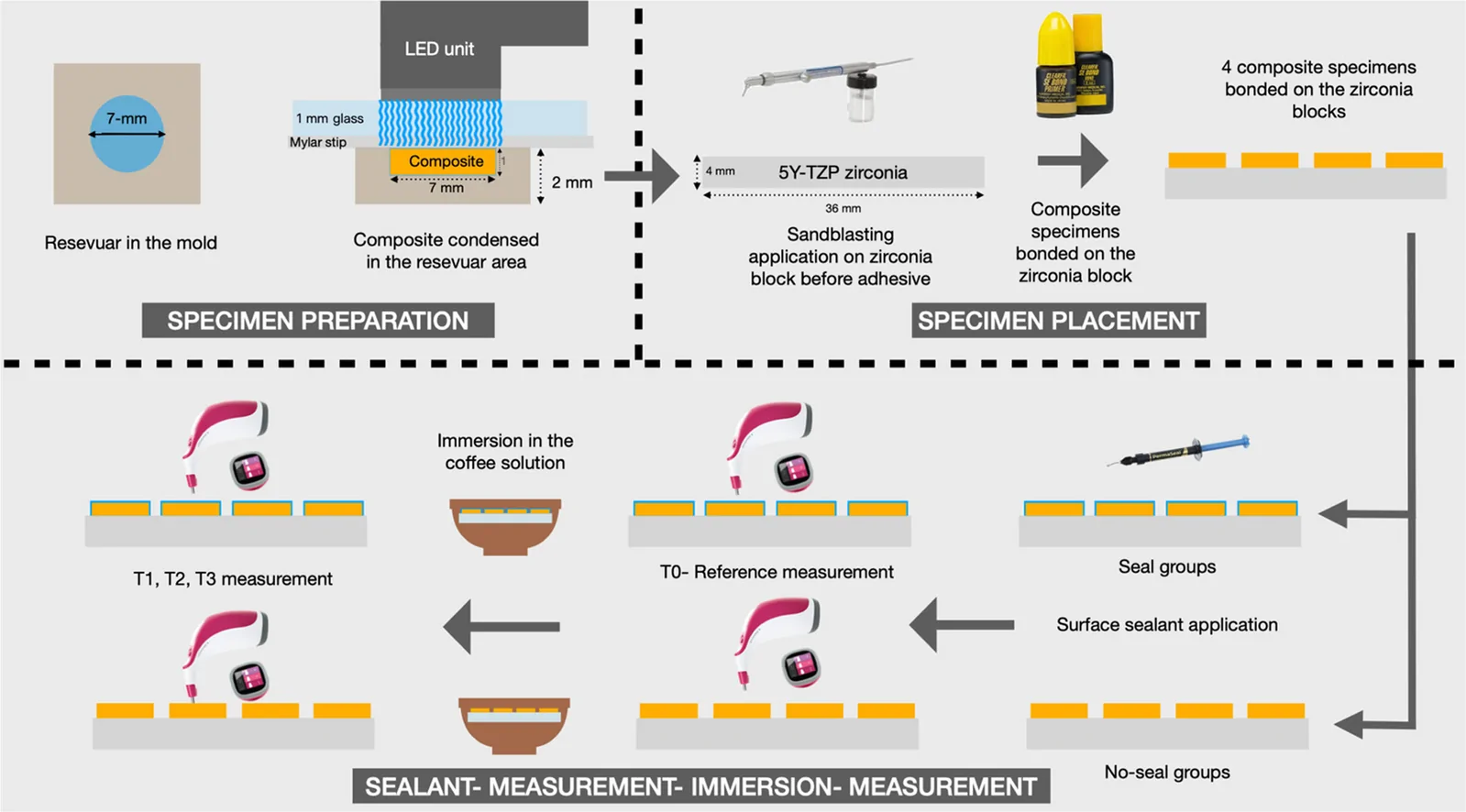
Experimental setup of the study

To simulate the attachment fabrication tray, a 2-mm-thick mold was fabricated using a light-transmitting, transparent silicone material (GC Exaclear, GC, Tokyo, Japan). This mold incorporated a reservoir featuring a cavity with a 7-mm diameter and a 1-mm depth (Fig. 1). For every group, respective composite material was applied into the prepared mold using a plastic instrument, after which a Mylar strip was placed over the composite. To standardize the light-curing distance and remove any excess composite material, a 1-mm-thick glass slide was positioned on top and manual pressure was applied to extrude the surplus material. The composites were initially polymerized for 20 s using LED curing unit (1200 mW/cm^2^, CuringPen-E, Eigthteeth, Changzhou, China). Following the removal of the glass slide, both the upper and lower surfaces were further polymerized for an additional 20 s each (Fig. 1).

The thickness of the obtained specimens was measured using a digital calliper (Mitutoyo Co, Kanagawa, Japan). To ensure that each specimen maintained a thickness of 1 ± 0.01 mm, the surfaces designated for bonding to blocks were, when necessary, abraded using wet silicon carbide papers with grits of 320, 600, 800, 1000 and 1200 subsequently.

All specimens were then immersed in distilled water at 37 °C for 24 h to ensure complete polymerization. After this period, the specimens were air-dried.

### Application of surface sealant

For the experimental groups designated for surface sealant (Permaseal, Ultradent Products, South Jordan, USA) application, surfaces of the attachment were conditioned according to the manufacturer's instructions. Specifically, 37% phosphoric acid (K-etchant, Kuraray, Osaka, Japan) was applied for 20 s, followed by rinsing with water for 20 s and air-drying for 20 s [20, 24]. After surface conditioning, sealant was applied on the attachment for 5 s using a specialized application tip (Black Micro FX, Ultradent, South Jordan, USA) with an oval brushing motion. After air-thinning from 10 cm for 20 s, the sealant was polymerized using the same LED curing unit for 40 s to ensure complete polymerization. Finally, to ensure complete polymerization, the specimens were immersed in distilled water and incubated at 37 °C for 24 h.

### Colour measurement

Colour analysis was conducted based on the CIE colour space. The L, a and b values of the specimens were determined using a dental spectrophotometer (VITA Easyshade V, VITA, Bad Sackingen, Germany). All colour measurements were performed in a dark room using a custom-made colour measurement box equipped with a 6500 K LED light and uniformly painted in neutral gray. The zirconia block was positioned at the center of the gray background, and for each specimen, three measurements were taken at the central region. The mean L*, a*, and b* values were then calculated. Prior to measuring each composite specimen, the spectrophotometer was calibrated in accordance with the manufacturer's instructions by placing it on the calibration block, where it was automatically calibrated. Measurements were carried out at four different time points: before immersion in the staining solution (T_0_), after 1 day of immersion in coffee solution (T_1_), after 3 days of immersion in coffee solution (T_2_) and after 6 days of immersion in coffee solution (T_3_) of immersion. Specimens were air-dried before each colour measurement.

### Discolouration process

A staining coffee solution was prepared by dissolving 10 g of instant coffee (Nescafe Classic, Nestlé) in 1000 mL of boiled water according to the manufacturer’s recommendation [14]. The specimens were immersed in 30 mL of the prepared coffee solution, which had been allowed to cool to room temperature, in a container that ensured complete submersion. Since clear aligners are typically removed during food and beverage consumption, the specimens in this study were exposed to the coffee solution without any aligner-like covering. During periods when measurements were not being conducted, the specimens were maintained in incubator at 37 °C in the coffee solution. The solution was refreshed daily, with the specimens being rinsed under running distilled water before being re-immersed in the new solution.

### Calculation of colour difference

The CIEDE2000 colour difference formula (∆E₀₀) was utilized to quantify the variation in colour values across different time points [30]. In the calculation of ∆E₀₀ values, the method described by Luo et al. [30] was followed, and the calculation table provided in the supplementary materials of the original publication was used. The measured CIE L*, a*, and b* values were entered into this table, which automatically performed the necessary transformations and generated the corresponding ∆E₀₀ values.

In the ∆E_00_ formula, ∆*L*, ∆*C* and ∆*H* denote the differences in value, chroma and hue, respectively, between the reference and experimental measurements. The rotation term (R_T_) is a function designed to account for the interaction between chroma and hue differences, particularly in the blue region of the colour space. Weighting functions (S_L_, S_C_ and S_H_) serve to adjust the total colour difference based on the spatial position of the colour pair within the *L*, *a* and *b* coordinates. Additionally, K_L_, K_C_ and K_H_ are parametric coefficients that act as correction factors for experimental conditions. The parametric coefficient ratio (K_L_: K_C_: K_H_) was set to 2:1:1 to ensure standardized calculations [30–32].

CIEDE2000 colour difference formula:

$$\Delta E_{00} = \sqrt{ \left(\frac{\Delta L'}{k_L S_L}\right)^2 + \left(\frac{\Delta C'}{k_C S_C}\right)^2 + \left(\frac{\Delta H'}{k_H S_H}\right)^2 + R_T \left(\frac{\Delta C'}{k_C S_C}\right) \left(\frac{\Delta H'}{k_H S_H}\right) }$$

For the colour difference calculations, T_0_ values were taken as the reference.

$$ \Delta \mathrm{E}_{00} T_1 = \mathrm{shade\ measurement\ at\ time\ } T_1 - \mathrm{reference\ measurement\ at\ time\ } T_0$$

$$\Delta \mathrm{E}_{00} T_2 = \mathrm{shade\ measurement\ at\ time\ } T_2 - \mathrm{reference\ measurement\ at\ time\ } T_0$$

$$\Delta \mathrm{E}_{00} T_3 = \mathrm{shade\ measurement\ at\ time\ } T_3 - \mathrm{reference\ measurement\ at\ time\ } T_0 $$

In addition, perceptibility and acceptability thresholds were considered for the interpretation of colour differences. According to Paravina RD et al., a ΔE₀₀ value below 0.8 was regarded as imperceptible, values between 0.8 and 1.8 as perceptible but clinically acceptable, and values above 1.8 as clinically unacceptable [33].

### Statistical analysis of colour difference

The normality assumption was assessed using the Shapiro–Wilk test and Q–Q plots for each experimental cell. The assumption of sphericity was evaluated using Mauchly’s test, and Greenhouse–Geisser correction was applied when this assumption was violated. Repeated measures 2-way ANOVA followed by post-hoc Tukey’s test was used for multiple comparisons. A computer software was used for the statistical analyses (JASP 0.19, JASP Team, 2024) (α = 0.05)(Table 3 and 4).

## Results

Regarding the period comparison, all groups showed a statistically significant trend in ∆E_00_ values following the sequence T_3_ > T_2_ > T_1`_ regardless of sealant application (Table 5).

**Table 3:** Between-subject effects from two-way repeated-measures ANOVA for the influence of composite type and surface sealant on colour change (ΔE₀₀)

	**Sum of Squares**	**df**	**Mean Square**	**F**	***p***	**η**^**2**^
Composite	371.123	3	123.708	78.584	<.001	0.282
Sealant	15.55	1	15.55	9,878	0.002	0.012
Composite ✻ Sealant	116.033	3	38.678	24,569	<.001	0.088
Residuals	138.531	88	1.574

**Table 4:** Within-subject effects from two-way repeated-measures ANOVA for the influence of time, composite type, and surface sealant on colour change (ΔE₀₀)

	**Sphericity Correction**	**Sum of Squares**	**df**	**Mean Square**	**F**	***p***	**η**^**2**^
Time	Greenhouse–Geisser	650.867	1.740	373.985	6156.998	<.001	0.494
Time ✻ Composite	Greenhouse–Geisser	12.64	5.221	2.421	39.856	<.001	0.01
Time ✻ Treatment	None	1.27	1.740	0.730	12.01	<.001	9.643 × 10–4
Time ✻ Composite ✻ Treatment	None	1.346	5.221	0.258	4.245	<.001	0.001
Residuals	None	9.303	153.151	0.061

**Table 5:** Statistical analysis of ∆E₀₀ mean colour difference in Seal and No-Seal groups: Between-group comparisons at individual time points and within-group comparisons across time

Surface Properties	**Group**	**∆E**_**00**_**T**_**1**_** Mean (SD)**	**∆E**_**00**_**T**_**2**_** Mean (SD)**	**∆E**_**00**_**T**_**3**_** Mean (SD)**
No-seal	AL-no seal	5.17 (0.50)^a,X^	7.46 (0.52)^a,Y^	9.02 (0.47)^a,Z^
	FL-no seal	6.34 (0.61)^b,X^	8.27 (0.90)^a,b,Y^	9.71 (0.79)^a,Z^
	SG-no seal	6.65 (0.81)^b,X^	8.09 (0.88)^a,Y^	9.89 (1.01)^a,Z^
	XT-no seal	6.61 (0.85)^b,X^	9.25 (0.99)^b,Y^	11.40 (0.99)^b,Z^
Seal	AL-seal	5.98 (0.540)^a,X^	7.98 (0.66)^a,Y^	9.38 (0.84)^a,Z^
	FL-seal	5.81 (0.28)^a,X^	7.74 (0.32)^a,Y^	9.07 (0.30)^a,Z^
	SG-seal	5.84 (0.55)^a,X^	7.76 (0.77)^a,Y^	9.11 (0.85)^a,Z^
	XT-seal	9.35 (1.06)^b,X^	11.84 (0.86)^b,Y^	13.57 (0.77)^b,Z^

Means sharing a superscript letter are not significantly different (*p >* 0.05)

Uppercase letters compare means in each row

Lowercase letters compare means in each column

Letters show comparisons within the seal and no-seal groups

*AL* Aligner Connect, *FL* Universal Flo, *SG* Estelite Sigma Quick, *XT* Transbond XT

**Scheme 1:**
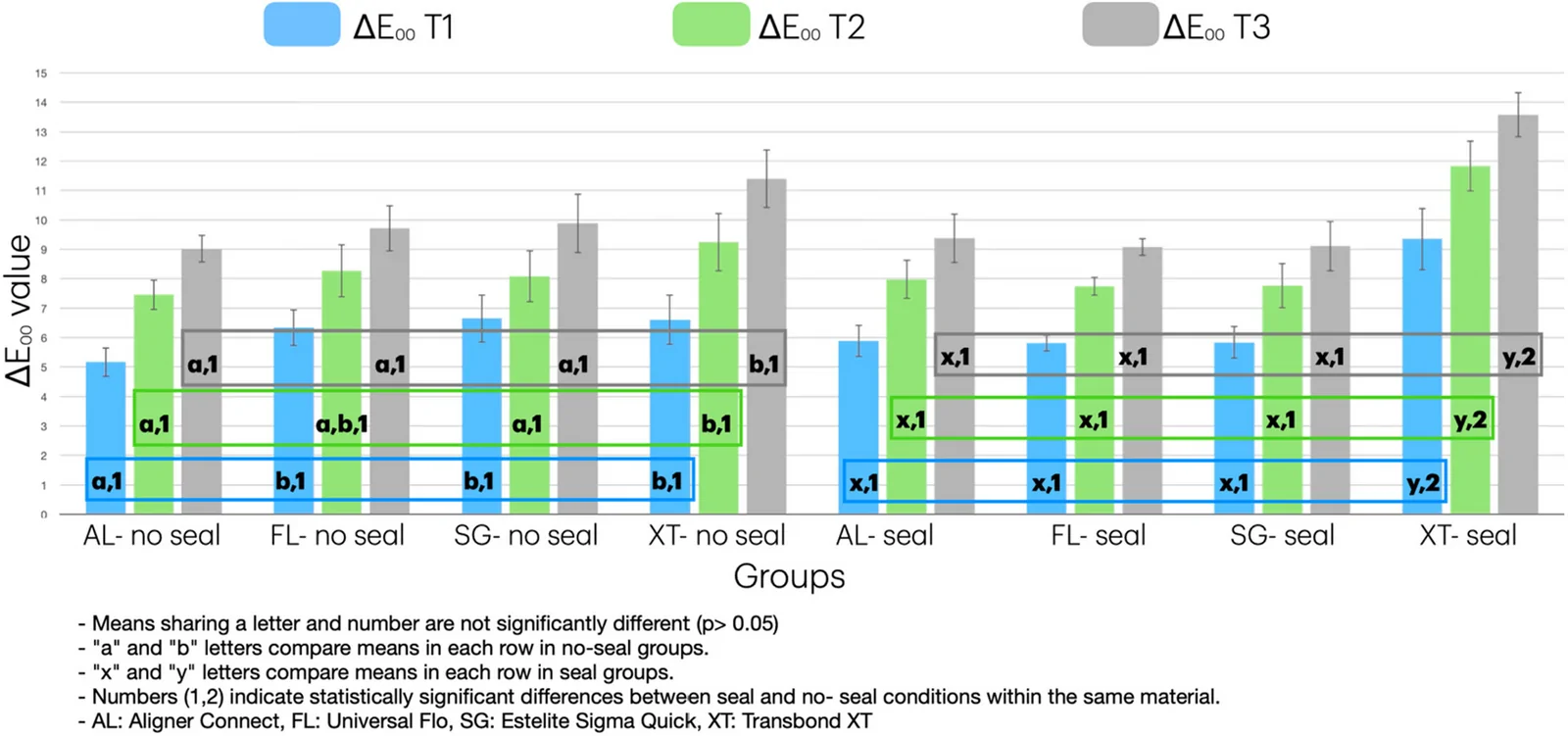
Mean ΔE_00_ values of the tested composite materials at T1, T2, and T3 with and without surface sealant

At T_1_, among the no-seal groups, aligner composite (AL-no seal) exhibited significantly lower ∆E_00_ values (∆E_00_T1: 5.17, ∆E_00_T2: 7.46, ∆E_00_T3: 9.02) than the those of the other resin materials (*p <* 0.05). Among the specimens treated with sealant, XT-seal group had significantly higher (∆E_00_T_1_: 9.35, ∆E_00_T_2_: 11.84, ∆E_00_T_3_: 13.57) ∆E_00_ values (*p <* 0.05) (Scheme 1).

At T_2_, among the no-seal groups, no significant differences were observed among the AL-no seal, FL-no seal and SG-no seal. The XT-no seal group recorded the highest ∆E_00_ value, though the difference between the XT-no seal and FL-no seal groups was not statistically significant (*p >* 0.05).

Surface sealant (Permaseal) application produced a statistically significant effect only in the orthodontic adhesive (∆E_00_XT-seal > ∆E_00_XT-no seal) group across all time points (Table 6).

**Table 6:** Comparisons of mean colour difference (∆E_00_) between no-seal and seal group per material

Resin Composite	Group	∆E_00_T_1_ Mean (SD)	∆E_00_T_2_ Mean (SD)	∆E_00_T_3_ Mean (SD)
Aligner Connect	AL-no seal	5.17 (0.50)^a^	7.46 (0.52)^a^	9.02 (0.47)^a^
	AL-seal	5.98 (0.540)^a^	7.98 (0.66)^a^	9.38 (0.84)^a^
Universal Flo	FL-no seal	6.34 (0.61)^a^	8.27 (0.90)^a^	9.71 (0.79)^a^
	FL-seal	5.81 (0.28)^a^	7.74 (0.32)^a^	9.07 (0.30)^a^
Estelite Sigma Quick	SG-no seal	6.65 (0.81)^a^	8.09 (0.88)^a^	9.89 (1.01)^a^
	SG-seal	5.84 (0.55)^a^	7.76 (0.77)^a^	9.11 (0.85)^a^
Transbond XT	XT-no seal	6.61 (0.85)^a^	9.25 (0.99)^a^	11.40 (0.99)^a^
	XT-seal	9.35 (1.06)^b^	11.84 (0.86)^b^	13.57 (0.77)^b^

Means sharing a superscript letter are not significantly different (*p >* 0.05)

Lowercase letters compare means of seal and no-seal specimens for each base material in the column

At T_3_, among the no-sealant groups, XT-no seal demonstrated the highest ∆E_00_ value (11.40) (*p <* 0.05) compared to other resin materials. Similarly, among sealed groups, XT-seal achieved the highest ∆E_00_ value (13.57) (*p <* 0.05).

**Scheme 2:**
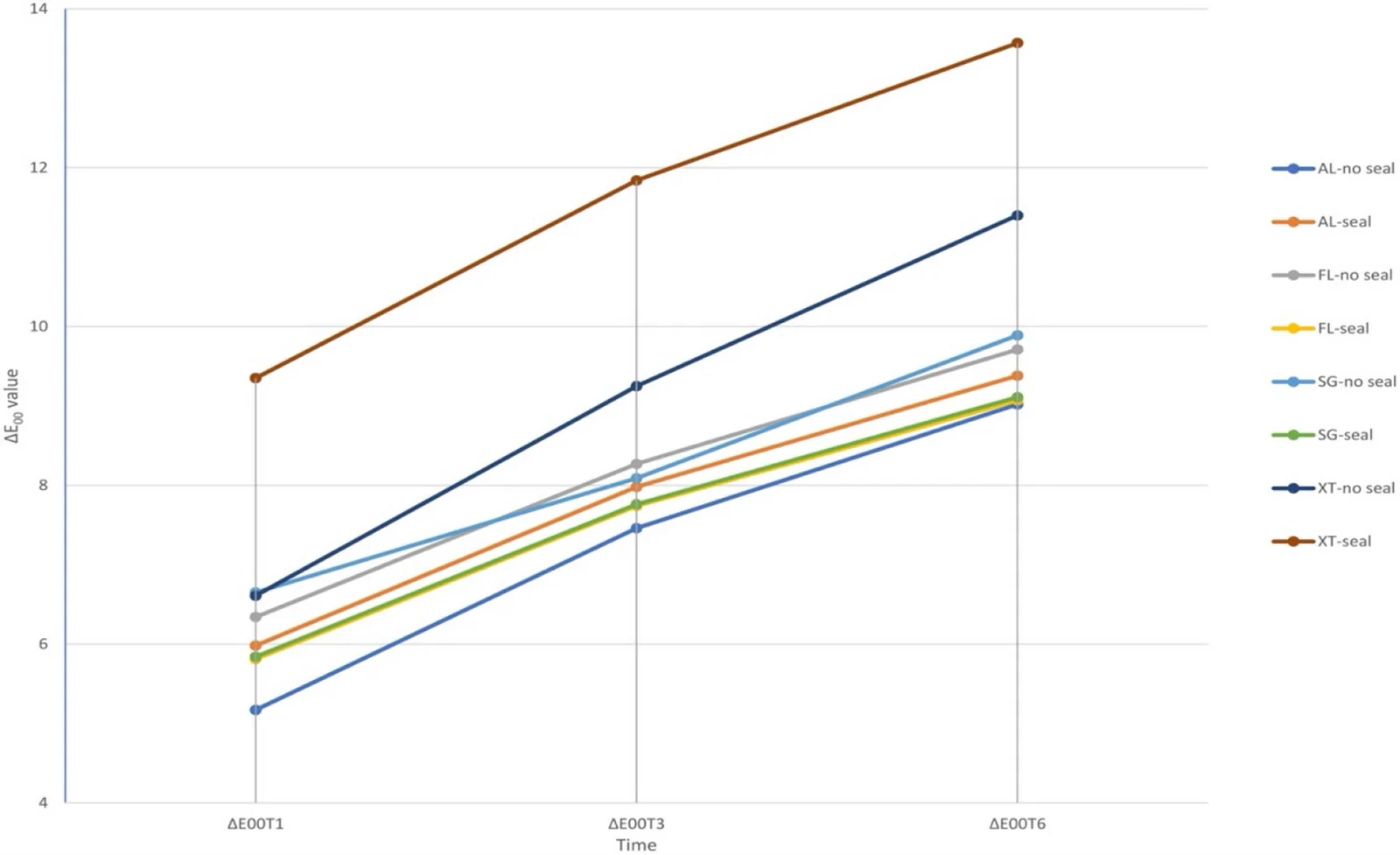
Changes ΔE_00_ according to time

Regarding the period comparison, all groups showed a statistically significant trend in ∆E_00_ values following the sequence T_3_ > T_2_ > T_1`_ regardless of sealant application (Scheme 2).

Regardless of sealant application, all resin composites demonstrated ∆E₀₀ values higher than the clinically acceptable threshold (1.8) at all times.

## Discussion

This study evaluated the colour stability of four resin composites used for orthodontic attachment fabrication, one paste-like restorative composite, one high-filler flowable restorative composite, one orthodontic adhesive, and one injectable aligner composite, after immersion in a coffee solution. The effect of a surface sealant on colour stability was also assessed. The sealant did not prevent discolouration; in fact, the sealed orthodontic adhesive (XT-seal) exhibited greater colour change than its unsealed counterpart (XT-no seal) at all time points. As the sealant provided no benefit in any group, the first null hypothesis was accepted. Since the unsealed orthodontic adhesive group showed significantly more discolouration than the AL-no seal, FL-no seal and SG-no seal groups, the second null hypothesis was partially rejected.

In dentistry, both visual and instrumental techniques are utilized for colour assessment [12]. Visual evaluation is subjective and lighting-dependent, thus instrumental measurements using spectrophotometers, spectroradiometers, or colorimeters are preferred for their reliability [34–38]. Therefore, VITA Easy Shade V spectrophotometer was used under standardised D65 illumination in a dark box.

The CIEDE2000 (∆E_00_) formula with parametric coefficients K_L_:K_C_:K_H_ = 2:1:1was adopted for colour difference calculations, as it provides results closer to human visual perception than the traditional CIELAB model [30–32, 39]. The ∆E_00_ values obtained in the present study exceeded the clinically acceptable threshold (> 1.8), indicating that the observed discoloration may be considered clinically relevant [33]. Despite statistical differences among groups, all materials exhibited clinically unacceptable colour changes. Although baseline colour differences existed among the materials the use of ΔE₀₀ calculations allowed standardised evaluation of colour changes over time within each material.

Coffee was chosen as the staining agent due to its high consumption rate and well-documented staining potential [14]. Based on average consumption patterns, six days of immersion corresponded to roughly six months of clinical exposure [40]. Therefore, measurements T1, T2 and T3 simulated one, three and six months, respectively. Considering the study results, the ΔE_00_ values increased proportionally with aging across all groups, showing a statistically significant difference in the order of T_1_ < T_2_ < T_3_. This progressive increase in discolouration may be attributed to the prolonged exposure to coffee, which could have led to surface degradation over time.

A substantial portion of the surface of orthodontic attachments remains in direct contact with the tooth in vivo and is thereby isolated from the oral environment and not directly exposed to staining agents [27, 41]. A study investigated the effect of surface exposure on discolouration by comparing specimens with all surfaces exposed to the staining solution (full-surface exposure) to those in which only one surface was isolated from exposure. The findings indicated that specimens with full-surface exposure exhibited significantly greater discolouration compared to the isolated ones [42]. In our study, to simulate the clinical application of attachments on tooth surfaces, the resin composite specimens were bonded onto zirconia blocks. This approach aimed to isolate the attachment surface in contact with the tooth from direct exposure to the staining solution, thereby mimicking routine clinical conditions.

Since natural teeth exhibit variability in form, thickness, colour and optical properties, zirconia blocks were used instead to eliminate these confounding factors and ensure standardization [27]. Zirconia is characterised by high colour stability and low susceptibility to staining, thereby reducing substrate-related colour interference during immersion procedures [43]. As colour measurements were obtained directly from the composite surface and the same substrate material was used for all groups, its influence on comparative outcomes is expected to be minimal, although it cannot be entirely excluded.

Surface sealants are designed to reduce water sorption and enhance colour stability [19]. However, the sealant (Permaseal) did not improve resistance and even increased discolouration in orthodontic adhesive (Transbond XT) (*p <* 0.05). This may relate to sealant’s Bis-GMA rich formulation, which promotes water uptake or degradation of TEGDMA under heat [43, 44].

Additionally, the sealant layer itself, due to its relatively low filler content and higher resin matrix composition compared to conventional resin composites, may be more susceptible to hydrolytic degradation over time, which has been associated with increased water sorption and colour instability in resin-based materials [21, 23]. Furthermore, the presence of an interfacial region between the sealant and the underlying composite may contribute to increased susceptibility to discoloration, particularly under prolonged exposure to aqueous staining media, as reported in studies evaluating surface sealants and composite interactions [20, 24]. However, this effect was not consistently observed across all materials in the present study, indicating that the influence of the sealant layer is likely material-dependent and may be related to the intrinsic properties of the underlying composite. This may explain the more pronounced discoloration observed in the orthodontic adhesive group.

A study reported numerically higher discolouration in composites treated with Permaseal; although the difference was not statistically significant compared untreated controls [24]. Similarly, another study evaluating various surface sealants under red wine exposure found that specimens treated with Permaseal higher colour change values, but again without statistical significance [23]. The resin-rich surface of attachments exposed to the oral environment, combined with the absence of polishing, likely increases their staining susceptibility. Previous studies have shown that attachments undergo clinically unacceptable colour changes (∆E_00_ > 1.8) over time, highlighting the need for improved surface protection strategies [15, 45–47].

The higher ∆E_00_ values observed in Transbond XT are in agreement with earlier finding showing that orthodontic adhesives undergo a greater colour change than other composites [11]. Although orthodontic adhesives are classified as resin composites, their formulation prioritizes bond strength under brackets rather than aesthetics. Likewise, its material composition, specifically, its high translucency, low pigment concentration, lower filler content and silane-coated fillers. Increased translucency and reduced pigment content have both been linked to greater discolouration [48, 49]. In addition, silane coating and its high water absorption capacity may further promote extrinsic staining [49]. Collectively, these factors may explain why Transbond XT is more prone to staining than other resin composites.

Manufacturers have developed high-viscosity flowable resin composites, such as GC Aligner Connect, specifically for orthodontic attachment fabrication. In clinical practice, however, both orthodontic and resin composites are used [45, 46]. In this study, the orthodontic attachment composite showed clinically unacceptable colour change from the first month (T_1_) but maintained the lowest AE_00_ values at all time point indicating comparatively better colour stability. This may be attributed to its hydrophobic fillers, which create a smooth, rigid surface after polymerisation and reduce susceptibility to staining.

Ercin et al. (2023) compared the discolouration of Aligner Connect with various restorative resin composites after 28 days found that, although it showed the lowest ΔE_00_ values, the difference was not statistically significant [15]. Similarly, in this study, after six days of immersion (simulating six months), no significant difference was observed between the AL group and the restorative resin composites FL and SG, regardless of surface sealant application. These findings align with the results of previous studies [15, 46].

One limitation of this study is the use of zirconia substrates instead of natural teeth, as the aim was to assess the intrinsic discolouration of the materials without the influence of tooth’s optical properties. A study replicating clinical conditions more comprehensively, including factors such as mechanical wear from brushing, repeated aligner insertion and temperature changes would yield more clinically relevant results. Future research should also investigate the effects of other surface sealants or surface treatments to better understand the long-term colour stability of resin composites used for orthodontic attachments. An additional limitation of this study is the absence of a negative control group, such as specimens stored exclusively in distilled water. Including such a group would have enabled a clearer distinction between colour changes resulting from intrinsic material ageing and those arising specifically from exposure to the coffee solution. Because the purpose of the present study was to evaluate staining behaviour under a single, clinically relevant challenge, all specimens were uniformly exposed to coffee without a non-staining control. Nonetheless, future studies incorporating negative controls would help better isolate the respective contributions of intrinsic degradation, water sorption and extrinsic staining in orthodontic attachment materials.

## Conclusion

Within the limitations of the study, the following conclusions can be drawn. However, direct clinical extrapolation of these findings should be approached with caution, as the present study was conducted under in vitro conditions.

1. The application of the surface sealant (Permaseal) did not improve the resistance of orthodontic attachments to discolouration from coffee, regardless of the composite material used.
2. The surface sealant (Permaseal) negatively affected the colour stability of the orthodontic adhesive (Transbond XT).
3. Coffee exposure caused clinically unacceptable discolouration in all tested composites, with the degree of staining increasing beyond the simulated one-month period.
4. The orthodontic adhesive resin (Transbond XT) showed the greatest discolouration among all materials tested. Clinicians should consider its aesthetic limitations when selecting materials for attachment fabrication.
5. The aligner composite (Aligner Connect) presented comparable or slightly better colour stability than restorative materials, supporting its clinical use for attachment fabrication.
6. Clinicians should advise patients with high aesthetic expectations that attachments may stain with frequent coffee consumption before treatment.

## Data Availability

The datasets used and/or analysed during the current study are available from the corresponding author on reasonable request.
